# A novel flow cytometry assay based on bacteriophage-derived proteins for *Staphylococcus* detection in blood

**DOI:** 10.1038/s41598-020-62533-7

**Published:** 2020-04-10

**Authors:** Susana P. Costa, Nicolina M. Dias, Luís D. R. Melo, Joana Azeredo, Sílvio B. Santos, Carla M. Carvalho

**Affiliations:** 1 Centre of Biological Engineering, University of Minho, Campus de Gualtar, 4710-057 Braga, Portugal; 2 International Iberian Nanotechnology Laboratory (INL), Av. Mestre José Veiga s/n, 4715-330 Braga, Portugal

**Keywords:** Bacteriophages, Infectious-disease diagnostics

## Abstract

Bloodstream infections (BSIs) are considered a major cause of death worldwide. *Staphylococcus* spp. are one of the most BSIs prevalent bacteria, classified as high priority due to the increasing multidrug resistant strains. Thus, a fast, specific and sensitive method for detection of these pathogens is of extreme importance. In this study, we have designed a novel assay for detection of *Staphylococcus* in blood culture samples, which combines the advantages of a phage endolysin cell wall binding domain (CBD) as a specific probe with the accuracy and high-throughput of flow cytometry techniques. In order to select the biorecognition molecule, three different truncations of the C-terminus of *Staphylococcus* phage endolysin E-LM12, namely the amidase (AMI), SH3 and amidase+SH3 (AMI_SH3) were cloned fused with a green fluorescent protein. From these, a higher binding efficiency to *Staphylococcus* cells was observed for AMI_SH3, indicating that the amidase domain possibly contributes to a more efficient binding of the SH3 domain. The novel phage endolysin-based flow cytometry assay provided highly reliable and specific detection of 1–5 CFU of *Staphylococcus* in 10 mL of spiked blood, after 16 hours of enrichment culture. Overall, the method developed herein presents advantages over the standard BSIs diagnostic methods, potentially contributing to an early and effective treatment of BSIs.

## Introduction

Bloodstream infections (BSIs) are severe diseases caused by the presence of microorganisms, mainly bacteria, in blood and are characterized by high morbidity and mortality^1,2^. The BSIs and associated organ dysfunctions (sepsis or septic shock) remain a life-threating disease due, partially, to the inability to rapidly detect and identify the responsible pathogens^3,4^.

*Staphylococcus* spp. are Gram-positive facultative anaerobic bacteria that frequently colonize the human body^5,6^. These pathogens are becoming increasingly resistant to antibiotics and are well-established in both community and healthcare environments, being commonly isolated in intensive care units (ICU)^6,7^. *Staphylococcus aureus* is a common cause of a variety of infections, from superficial skin infections to life-threatening diseases, including necrotizing pneumonia^8^, infective endocarditis^9^ and BSIs^10^. Coagulase-negative staphylococci (CoNS) have also been described as harmful to humans, causing several infections, particularly in patients with implanted medical devices^6^.

The empirical antibiotic therapy remains the standard of BSIs treatments^11^ and its correct use within the first hour after the recognition of the BSI is recommended by the Surviving Sepsis Campaign Guidelines^11^ and was reported as having a great impact on the patient survival rate^12^. Nevertheless, the extensive use of broad-spectrum antibiotics and the large number of patients having negative blood culture samples and thus receiving unnecessary antibiotic treatment, are important contributors to the increase of antimicrobial resistance^13–15^. Thus, sensitive, rapid, cost-efficient and specific detection of pathogens in blood, followed by antimicrobial testing, is critical to de-escalate empirical antibiotic therapy and decrease the negative impact of BSIs^2,14,16^.

Blood cultures remain the reference standard for the detection of bacteria causing sepsis^17^. Generally, blood samples are collected and aseptically inoculated in bottles with specific media for aerobic and anaerobic microorganisms. These bottles are then incubated either in manual or in automatic systems that continuously monitor microbial growth^17^.

The conventional culture methods for diagnosis of BSIs involve sub-culturing and Gram staining upon blood-culture positivity, followed by phenotypic methodologies for bacterial identification and antibiotic susceptibility testing. These procedures can be accurate and reliable but are laborious and time-consuming^18^. In the last decade, other detection techniques have emerged as alternatives to conventional culture methods for the detection of BSIs, directly from positive blood cultures or from whole blood, and have been improved the time needed for pathogen identification. These include the Polymerase Chain Reaction (PCR)^19,20^, Peptide Nucleic Acid Fluorescence *In Situ* Hybridisation (PNA-FISH)^21,22^, Matrix-Assisted Laser Desorption Ionization Time-of-Flight Mass Spectrometry (MALDI-TOF MS)^23^ and DNA microarrays^24^. However, these methods present some drawbacks, namely: PCR-associated amplification problems (such as PCR inhibitors)^25^, unspecific hybridization, which can be caused by the human DNA interference with primers and probes^25,26^, infidelity in DNA replication, interference of non-microbial material^17,25^, limited number of available probes^18^, the results obtained are complex and difficult to interpret^26^, and are unable to distinguish between live and dead cells leading to the occurrence of false positives^25,26^. Moreover, pathogen detection directly from blood samples remains a challenge due to the innumerable blood components that can interfere in the analysis^25,26^ and to the low bacterial load normally present in the blood from patients with BSIs (1 to 100 CFU mL^−1^)^26,27^. Consequently, most of the detection methods for BSIs are dependent on blood cultures to increase the number of pathogens before the diagnostic test can be conducted^17^.

A promising approach for bacterial detection is the use of bacteriophages (phages) or phage-derived proteins as specific probing elements in conjugation with measurement techniques or biosensors. Phages are viruses that infect bacteria with high host specificity^28^. At the end of their life cycle, phages produce enzymes, called endolysins, to degrade the bacterial cell wall for the release of progeny virions. These proteins have been considered valuable tools to detect and control bacterial infections^29–33^. Endolysins from phages infecting Gram-positive bacteria present a modular structure composed of at least one enzymatic catalytic domain (ECD) and one cell binding domain (CBD)^29^. Most staphylococcal phage endolysins have three distinct domains: two ECDs, namely an N-terminal cysteine histidine-dependent amidohydrolase/peptidase (CHAP) domain and a central N-acetylmuramoyl-L-alanine amidase (Ami_2 or Ami_3) domain; and a C-terminal bacterial src-homology 3 (SH3b) domain as CBD^34,35^. Generally, the C-terminal CBD is responsible for directing the enzyme to its substrate by recognizing specific ligands on the bacterial peptidoglycan or other cell wall-associated molecules with extreme specificity and affinity^30,36^. Therefore, CBDs have been used as probing elements for the rapid detection or concentration of bacteria^32,37,38^, mainly foodborne pathogens^31^. The CBDs binding spectra are usually broader than the host ranges of the corresponding phage and can encompass entire bacterial genera^39^ and their binding affinity to bacterial cells is equal or even higher than antibodies^36^. Moreover, phage proteins have shown increased stability to temperature and pH variations, less propensity to aggregation and a faster, easier and less expensive production in comparison with antibodies^40,41^.

Flow cytometry is a multiparametric high-throughput technique which enables the detection and quantification of cells or particles individually in a flow, allowing a rapid and effective analysis of their physical and chemical properties within a population^42^. The cells are often labelled with fluorescent markers, therefore allowing the discrimination of different populations in a sample^42^. In the past years, this technique has been widely used for pathogen detection purposes, although the assays that have been described present some drawbacks, such as being laborious and expensive^43^ or lack the ability to detect pathogens in biological samples^44,45^.

In this study, we describe a novel flow cytometry assay based on phage-derived proteins for the detection of *Staphylococcus* in blood. For this, we exploited different domains at the C-terminus of the *S. aureus* phage endolysin E-LM12^46^ (Amidase, SH3 and Amidase+SH3) in order to select the truncation that presents the higher binding affinity and specificity for *Staphylococcus* spp. to be used as a specific probe in the detection assay. To the best of our knowledge, this is the first detection method described in the literature, in which a CBD was used as a recognition molecule in a flow cytometry assay, enabling the sensitive and specific detection of *Staphylococcus* in blood.

## Results

### Bioinformatics analysis of E-LM12

**Figure 1:**
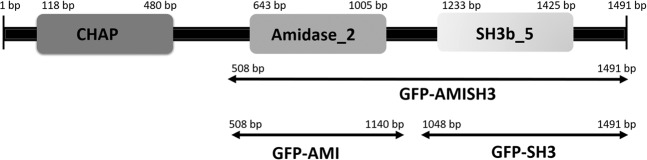
Prediction of E-LM12 endolysin domains and schematic representation of the different fragments that were cloned. The endolysin contains a N-terminal CHAP catalytic domain, a central Amidase-2 domain, and a C-terminal SH3b_5 binding domain.

After identifying the endolysin gene E-LM12 (gp159) from the *S. aureus* vB_SauM-LM12 phage genome^46^, a BLAST analysis revealed that E-LM12 shows high similarity with endolysins from several staphylococcal phages, including *Staphylococcus* phage MCE-2014 (100% coverage, 95% identity)^47^, *Staphylococcus* phage phiIPLA-RODI (100% coverage, 94% identity)^48^ and *Staphylococcus* phage vB_SauM_0414_108 (99% coverage, 95% identity)^49^. Also, no similarity was found with endolysins from phages infecting other bacterial genera. The search for functional domains predicted the existence of three domains (Fig. 1): an N-terminal CHAP catalytic domain, a central Amidase_2 catalytic domain and a C-terminal SH3b_5 cell binding domain^46^. In the same region of the Amidase_2, a peptidoglycan recognition protein (PGRP) domain was also predicted.

### Functional analysis of the E-LM12 endolysin C-terminus

**Figure 2:**
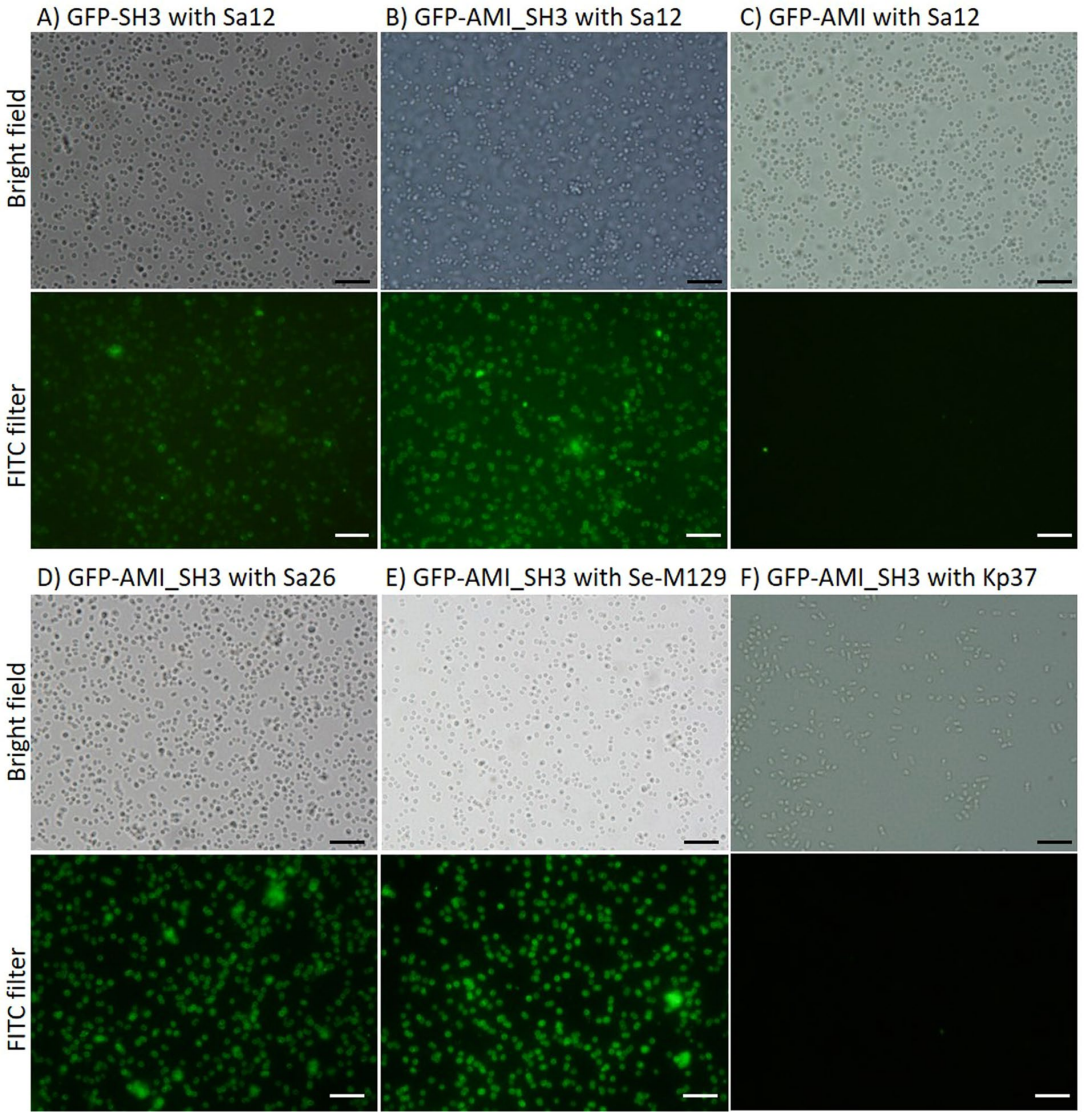
Fluorescence microscopy images of the different domains of E-LM12 endolysin and specificity assay of GFP-AMI_SH3. *S. aureus* Sa12 cells incubated with: GFP-SH3 (**A**), GFP-AMI_SH3 (**B**) and GFP-AMI (**C**). Assessment of the binding affinity of GFP-AMI_SH3 protein after incubation with: *S. aureus* Sa26 (**D**), *Staphylococcus epidermidis* M129 (**E**) and *Klebsiella pneumoniae* 37 (**F**). Observations were made in bright field and under FITC filter with the same exposure time to detect the presence/absence of fluorescing cells. Scale bar represents 10 µm.

Three different fragments from the C-terminus of the E-LM12 endolysin^46^ were cloned (AMI, SH3 and AMI_SH3) fused to a green fluorescent protein (GFP), originating the recombinant proteins GFP-AMI, GFP-SH3 and GFP-AMI_SH3 (Fig. 1). The binding ability of these proteins was assessed by epifluorescence microscopy through the observation of cells emitting green fluorescence. The results showed that the fusion proteins GFP-SH3 and GFP-AMI_SH3 were capable of binding to *S. aureus* cells, with the GFP-AMI_SH3 clearly displaying a higher binding ability than the GFP-SH3, as observed by the intensity of the green fluorescence at the microscope (Fig. 2A,B). Conversely, no fluorescent cells were observed when the GFP-AMI protein (Amidase domain) was added to the host bacteria (Fig. 2C).

#### Specificity and sensitivity of GFP-AMI_SH3 and comparison with the lytic activity of LM12 phage and E-LM12 endolysin

Considering that the GFP-AMI_SH3 revealed a higher binding ability to *S. aureus* cells, the binding spectrum and specificity of this protein was evaluated by epifluorescence microscopy. For this, the protein was incubated with strains of *Staphylococcus* spp. and other Gram-positive and Gram-negative bacteria (Table 1). Simultaneously, bacterial cell samples without GFP-AMI_SH3 and the GFP individually were used as negative controls. All the strains tested and the respective results are summarized in Table 1.

**Table 1:** Lytic spectrum of LM12 phage and binding affinity of GFP-AMI_SH3 protein obtained by flow cytometry and epifluorescence microscopy assays.

Species	Strain	LM12 Phage Lysis	GFP-AMI_SH3 Affinity	
		Spot lysis test	EFM	FC
***Staphylococcus*** **species**				
*Staphylococcus aureus*	Sa12 (host)	+	+	+
	C017	+	+	+
	C060	+	+	+
	C101	+	+	+
	C117	+	+	+
	C276	+	+	+
	C411A	+	+	+
	C557	+	+	+
	Sa1	+	+	+
	Sa2	+	+	+
	Sa3	LFW	+	+
	Sa26	LFW	+	+
*Staphylococcus capitis*	SECOM052A^85^	+	+	+
*Staphylococcus epidermidis*	IE186^86^	+	+	+
	PT12003^87^	+	+	+
	RP62A^85^	LFW	+	+
	SECOM020A.1^85^	+	+	+
	M129^88^	+	+	+
*Staphylococcus equorum*	SECOM060A^85^	LFW	+	+
*Staphylococcus haemolyticus*	SECOM065A.1^85^	−	+	+
*Staphylococcus hominis*	SECOMM11^85^	LFW	+	+
*Staphylococcus warneri*	SECOMF16^85^	+	+	+
Other Gram-positive bacteria				
*Bacillus cereus*	1	−	−	−
*Bacillus subtilis*	DSMZ10	−	−	−
*Enterococcus faecalis*	CECT 184	−	−	−
	LMV-0-34	−	−	−
	LMV-0-39	LFW	±	±
*Enterococcus faecium*	LMV-0-42	LFW	±	±
	CECT 410	−	−	−
*Listeria monocytogenes*	CECT 938	−	−	−
*Streptococcus pneumoniae*	R6st	−	±	±
Gram-negative bacteria				
*Acinetobacter baumannii*	RUH 134	−	−	−
	13	−	−	−
*Citrobacter freundii*	1	−	−	−
	36	−	−	−
*Enterobacter aerogenes*	CECT 684	−	−	−
*Escherichia coli*	2	−	−	−
	5	−	−	−
*Klebsiella pneumoniae*	35	−	−	−
	37	−	−	−
*Pseudomonas aeruginosa*	PA3	−	−	−
	PA4	−	−	−

LFW - lysis from without; EFM – epifluorescence microscopy; FC – flow cytometry.

EFM: (+) means that most of the cells were visualized as being green fluorescent; (±) means that few cells were fluorescent; (−) means that no fluorescent cells were visualized.

FC: (+) means that ≥90% of the cell population was recorded in the positive quadrant; (±) means that ≤10% of the cell population was recorded in the positive quadrant; (−) means that just a neglectable number of the cell population was in the positive quadrant (≤5%).

The results demonstrated that the GFP-AMI_SH3 binding spectrum covered all staphylococcal strains tested including 12 *S. aureus* (representative example in Fig. 2D) and 10 other staphylococcal species (example in Fig. 2E), including CoNS. Some weak binding was visualized for *Enterococcus faecium* LMV-0-42, *Enterococcus faecalis* LMV-0-39 and *Streptococcus pneumoniae* R6st (see examples in Supplementary Fig. S1 online). In these cases, the fluorescence intensities were lower than the obtained for *Staphylococcus* species and its distribution was not homogeneous along the bacterial cell wall surfaces, with some intense dots randomly distributed along the cells. In contrast, no fluorescent cells were detected on the other 17 non-*Staphylococcus* spp. strains tested with GFP-AMI_SH3, including Gram-positive and Gram-negative bacteria (example in Fig. 2F). Also, control experiments with unlabelled cells and GFP individually did not display green fluorescence.

The binding affinity of GFP-AMI_SH3 was compared with the lytic activity of LM12 phage using the same strains (Table 1). The phage showed a lytic effect against all *Staphylococcus* spp. strains tested, presenting “lysis from without” (LFW)^50^ on five *Staphylococcus* strains, and was unable to lyse the *S. haemolyticus* strain. The phage also revealed LFW when tested against *E. faecalis* LMV-0-39 and *E. faecium* LMV-0-42 but did not lyse the other non-staphylococcal strains (Table 1). The E-LM12 endolysin showed a lytic activity similar to the binding affinity of GFP-AMI_SH3, being able to lyse all the *Staphylococcus* strains tested (Table 1) when concentrations equal to or higher than 5 µM were used.

### Flow cytometry assays

#### Exploitation of the C-terminus E-LM12 endolysin domains as recognition molecules

In order to select the recognition molecule to be used on the flow cytometry assays, the three fusion proteins (GFP-AMI, GFP-AMI_SH3 and GFP-SH3) were incubated with *S. aureus* cells and samples were analysed using a flow cytometer equipped with a laser capable of exciting the GFP. The cells that were decorated with the proteins emitted green fluorescence, which was detected on the channel FL1, therefore allowing for the discrimination between labelled and unlabelled cells.

**Figure 3:**
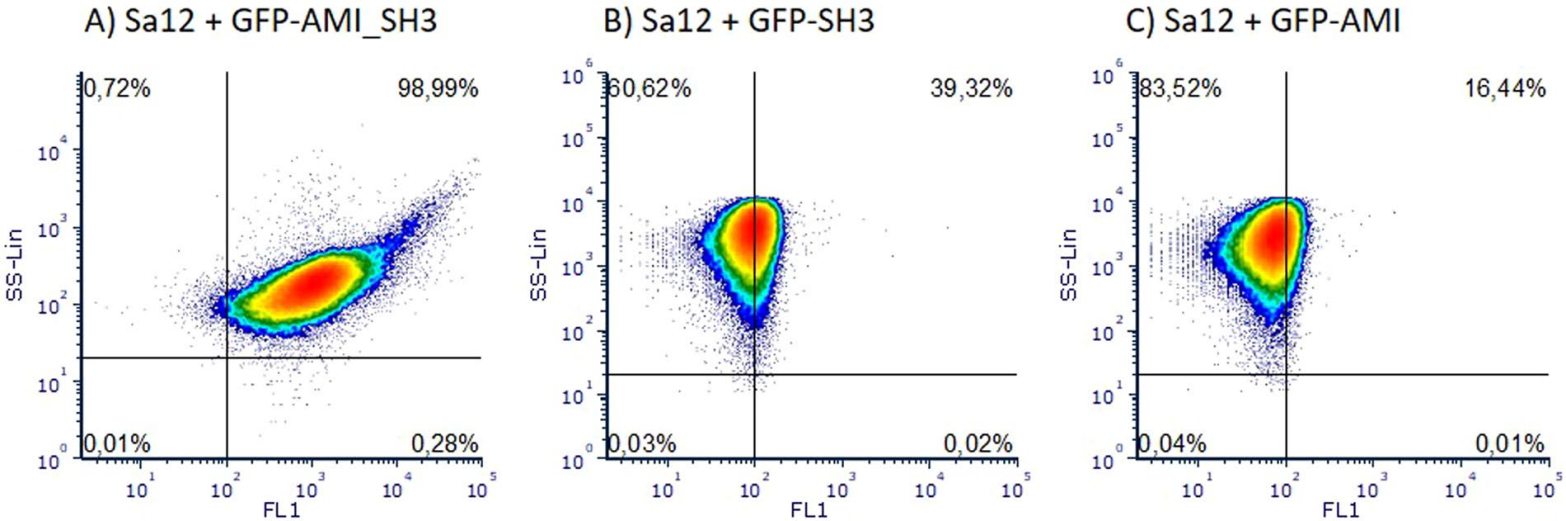
Flow cytometry analysis of the different domains of E-LM12 endolysin. Dot plots showing side scattering (SS) and green fluorescence (FL1) of *S. aureus* Sa12 cells after incubation with: GFP-AMI_SH3 (**A**), GFP-SH3 (**B**) and GFP-AMI (**C**).

The results indicate that the GFP-AMI_SH3 showed the highest binding efficiency, decorating most of the *S. aureus* cells (about 99% of the population was in the positive quadrant) (Fig. 3A) when compared with the GFP-SH3 that stained only 39% of the population (Fig. 3B). Moreover, the intensity of the green fluorescence (FL1) of the positive population when using the GFP-AMI_SH3 protein was much higher compared with that using the GFP-SH3 protein, corroborating the results of the fluorescence microscopy assays. Conversely, the GFP-AMI revealed a negligible binding (about 16% of the population) (Fig. 3C), which was probably due to some sample residues or free protein that was not completely washed since no green fluorescent cells were detected by fluorescence microscopy (Fig. 2C).

Considering that the GFP-AMI_SH3 revealed a higher binding ability to *S. aureus* cells than the other fragments tested, this protein was chosen for further experiments.

#### Evaluation of detection parameters

Several detection parameters were assessed on the flow cytometry assays, using the GFP-AMI_SH3 as a recognition molecule. These comprised the specificity and sensitivity against staphylococcal and other Gram-positive and Gram-negative strains (Table 1) and the detection limit.

**Figure 4:**
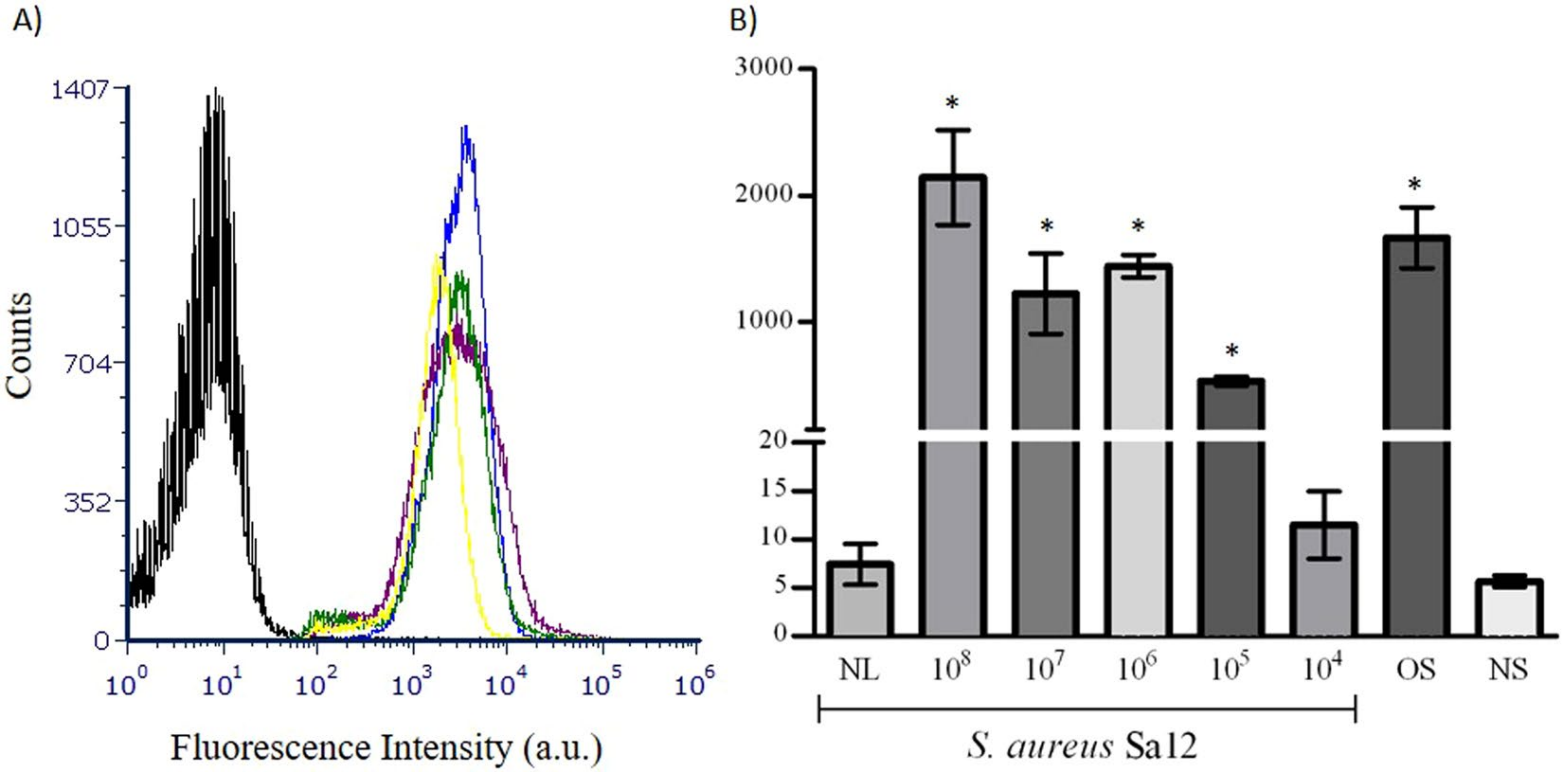
Graphical representation of the specificity and detection limit of the flow cytometry assays. (**A**) Overlay of the different histograms obtained by flow cytometry after incubation of GFP-AMI_SH3 with different bacterial strains. In black: *A. baumannii* 13; blue: *S. aureus* Sa12; green: *S. warneri* SECOMF16; purple: *S. aureus* Sa26; and yellow: *S. epidermidis* RP62A. (**B**) Mean of the fluorescence intensity (in arbitrary units (a.u.)) after incubation of GFP-AMI_SH3 with: *S. aureus* Sa12 at different concentrations (10^4^ CFU mL^−1^ to 10^8^ CFU mL^−1^); “OS”- Other *Staphylococcus* - *S. warneri* SECOMF16, *S. aureus* Sa26 and *S. epidermidis* RP62A (10^8^ CFU mL^−1^); and “NS” - non-staphylococcal bacteria- *A. baumannii* 13, *K. pneumoniae* 35 and *P. aeruginosa* PA3 (10^8^ CFU mL^−1^). Error bars represent standard deviations from three independent experiments performed in duplicate. *Statistically significant (p value <0.05) differences of fluorescence intensity mean values from the unlabelled *S. aureus* Sa12 (NL).

The assays clearly demonstrated that the developed method was able to specifically detect all the *Staphylococcus* spp. strains tested, including *S. aureus*, *S. epidermidis*, *S. equorum*, *S. haemolyticus*, *S. hominis* and *S. warneri* (Table 1). As depicted in Fig. 4A, the histograms of the *Staphylococcus* spp. strains were in the right side with fluorescence values beginning in 100 a.u. while the histogram of a non-staphylococcal bacteria was shifted to the left. Moreover, the fluorescence intensity means were similar for *S. aureus* Sa12 and other staphylococcal strains (means of 2,142.4 ± 373.2 a.u. and 1,665.4 ± 241.2 a.u., respectively), but were considerable different from the non-*Staphylococcus* strains tested (mean of 7.7 ± 4.4 a.u.) (Fig. 4B). Also, mean fluorescence intensity from labelled *Staphylococcus* spp. strains was around 200-fold higher than the value obtained from unlabelled *S. aureus* cells (NL), used as negative control (mean of 7.4 ± 2.1 a.u.) (Fig. 4B).

For the determination of the detection limit, different concentration of *S. aureus* Sa12 cells (ranging from 1 × 10^4^ CFU mL^−1^ to 1 × 10^8^ CFU mL^−1^) incubated with GFP-AMI_SH3 were analysed through the flow cytometer. Based on these assays, the minimum concentration of bacteria for which it was possible to consider a positive signal, significantly different from the controls (unlabelled cells and labelled non-*Staphylococcus* strains), was 10^5^ CFU mL^−1^ (Fig. 4B).

#### **Detection of*S. aureus*in spiked blood samples**

The flow cytometry assay was adapted and optimized in order to assess its ability to specifically detect *S. aureus* in artificially seeded blood samples. For this, as a proof-of-concept, horse blood was inoculated with 1 to 5 CFU mL^−1^ of *S. aureus* Sa12, followed by an enrichment for 16 hours. Samples were processed following an optimized procedure, which included treatment with water to deplete the red blood cells followed by centrifugations. Samples were then incubated with GFP-AMI_SH3 and analysed by flow cytometry. As a negative control, blood was mixed with medium without bacteria and submitted to the same procedure as the spiked samples, to evaluate unspecific binding of the fusion protein. In addition, unlabelled *S. aureus* Sa12 and a blood sample without protein were used as controls for the autofluorescence of bacterial cells and blood components, respectively.

**Figure 5:**
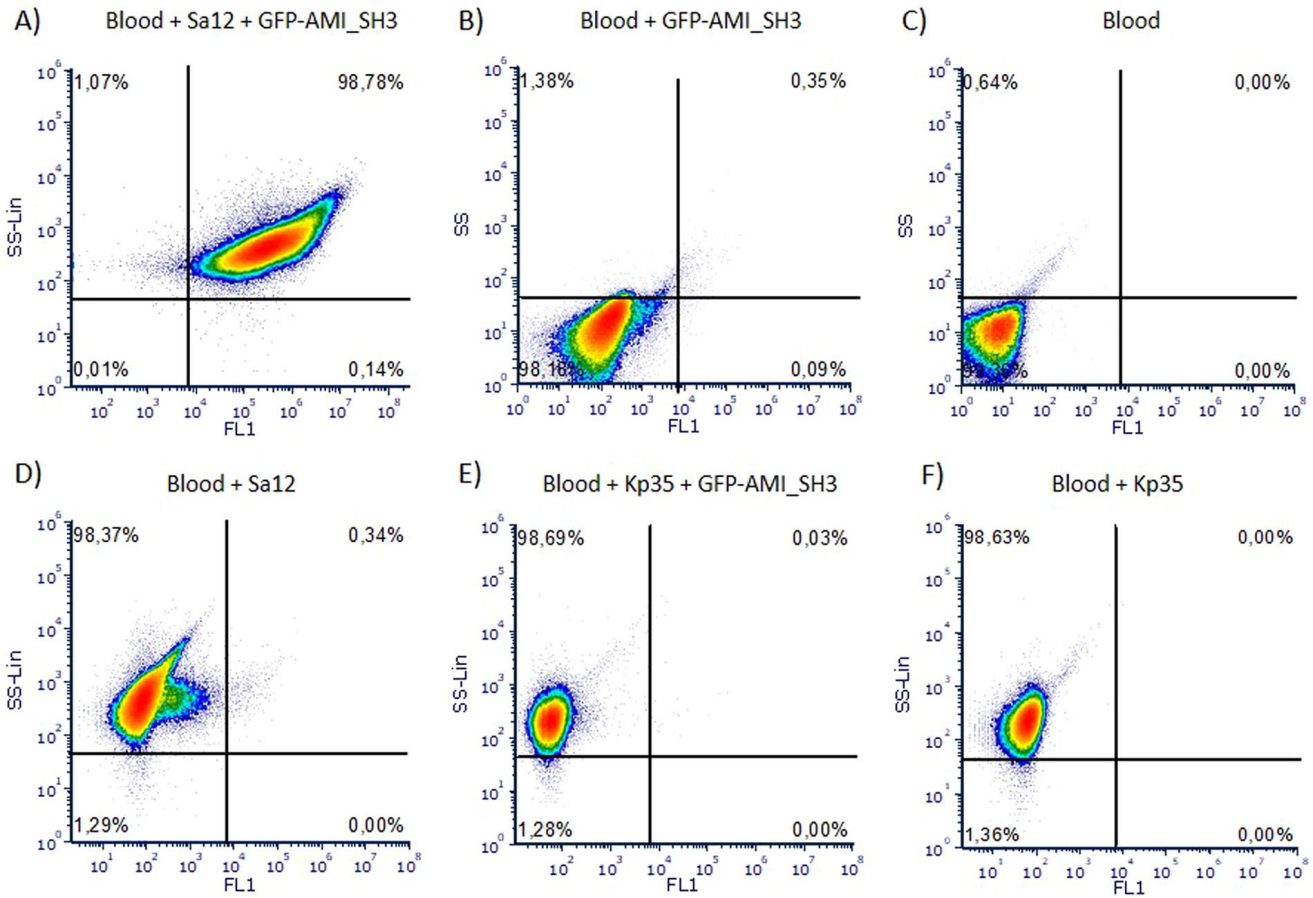
Graphical representation of blood samples analysis by flow cytometry. Representative dot plots showing side scattering (SS) and green fluorescence intensity (FL1) of: blood culture with *S. aureus* Sa12 decorated with GFP-AMI_SH3 (**A**); blood with GFP-AMI_SH3 (**B**); blood (**C**); blood culture with *S. aureus* Sa12 (**D**); blood culture with *K. pneumoniae* 35 (Kp35) incubated with GFP-AMI_SH3 (**E**); and blood culture with *K. pneumoniae* 35 (Kp35) (**F**).

The results represented in Fig. 5 revealed that the GFP-AMI_SH3 combined with flow cytometry was successfully able to detect *S. aureus* cells in blood cultures. The protein was capable of decorating *S. aureus* cells, allowing the detection of about 99% of that population (Fig. 5A). On the contrary, blood samples incubated with GFP-AMI_SH3 showed a negligible signal (about 0.35%) (Fig. 5B). In addition, the blood samples without GFP-AMI_SH3 (Fig. 5C) and the unlabelled *S. aureus* cells (Fig. 5D) did not emit a detectable fluorescence.

A specificity test was performed with *K. pneumoniae* 35 as negative control bacteria, following the same procedure used for *S. aureus* cells. The biparametric dot plot is displayed in Fig. 5E, demonstrating that only a minor number of events were recorded as positives (0.03% of the population), which was similar to the unlabelled *K. pneumoniae* cells (Fig. 5F). Moreover, since under the epifluorescence microscopy, GFP-AMI_SH3 showed a slight binding to *E. faecalis* LMV-0-39 and *E. faecium* LMV-0-42, these bacteria were further spiked in blood and the fluorescence was measured by flow cytometry. The results demonstrated that only a low percentage of the bacterial population (<5%) was plotted as positive (see Supplementary Fig. S2 online), which can be interpreted as a negligible signal.

**Figure 6:**
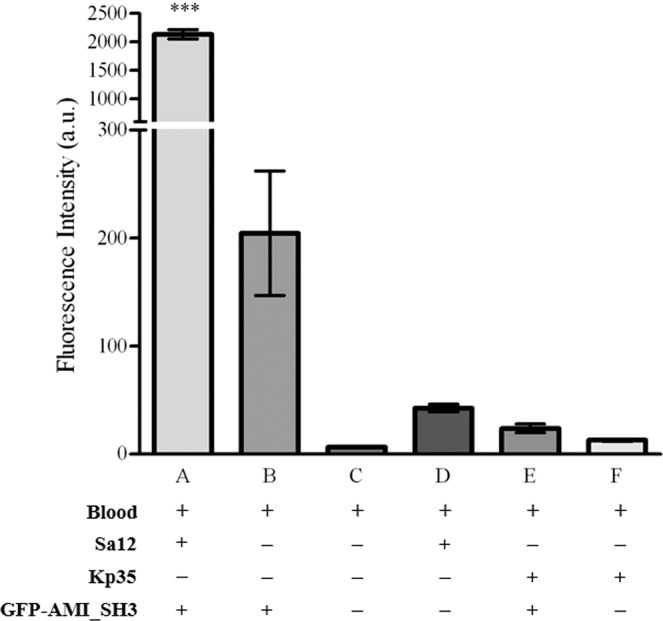
Graphical representation of the mean of the fluorescence intensity of blood samples analysed by flow cytometry. Mean of the fluorescence intensity of: blood culture with *S. aureus* Sa12 decorated with GFP-AMI_SH3 (**A**); blood with GFP-AMI_SH3 (**B**); blood (**C**); blood culture with *S. aureus* Sa12 (**D**); *K. pneumoniae* 35 blood culture with GFP-AMI_SH3 (**E**); and *K. pneumoniae* 35 blood culture (**F**). All the strains were at 10^8^ CFU mL^−1^, after a 16 hours inoculum (approximately 1 CFU mL^−1^) in blood. Error bars represent standard deviations from three independent experiments. Statistically highly significant differences (p < 0.001) of pairwise comparisons between the mean of the fluorescence intensity of labelled *S. aureus* Sa12 cells among all possible conditions (***) were determined by one-way ANOVA with a post-hoc Tukey’s multiple comparisons test.

Overall, the fluorescence signal obtained from the *S. aureus* samples decorated with GFP-AMI_SH3 was clearly distinguishable from the background signal (Fig. 6). The mean fluorescence intensity of labelled *S. aureus* cells (Fig. 6A) was 10-fold higher than the mean value obtained from the blood samples with GFP-AMI_SH3 (Fig. 6B) (means of 2,136.8 ± 145.6 and 204.6 ± 99.8 a.u., respectively). The weak fluorescence signal measured on the labelled blood samples can be attributed to the presence of free protein that has not been completely washed or to the incomplete lysis of erythrocytes which are known to have some autofluorescence^43^. The blood sample (Fig. 6C) and the unlabelled *S. aureus* cells (Fig. 6D) displayed minimal fluorescence intensity means (6.7 ± 4.4 and 42.8 ± 6.2 a.u., respectively). Also, the signal obtained for *K. pneumoniae* (Fig. 6E) was significantly (p < 0.001) lower than the one obtained for *S. aureus* (Fig. 6A) and similar to the mean value obtained from unlabelled *K. pneumoniae* cells (Fig. 6F). Pairwise multiple comparisons were performed between all tested samples, and the results revealed that the mean fluorescence intensity of the signal emitted from *S. aureus* Sa12 cells labelled with GFP-AMI_SH3 was significantly different (p < 0.001) comparing with the mean value of fluorescence signal from the control samples namely blood, blood with GFP-AMI_SH3, unlabelled *S. aureus* and *K. pneumoniae* cells, and *K. pneumoniae* with GFP-AMI_SH3. Moreover, no statistically significant differences (n.s) were obtained when comparing these control samples (Fig. 6).

## Discussion

BSIs and sepsis are the leading causes of human mortality among hospitalized patients and thus have a great impact on healthcare systems. Staphylococci are frequently isolated bacteria from BSIs and therefore their rapid identification from blood would allow the early diagnosis of a possible septicaemia^7,10^.

In this study, a new endolysin-based flow cytometry assay was developed for the early detection of *Staphylococcus* in blood. Firstly, different domains at C-terminus of *S. aureus* E-LM12 endolysin were cloned fused to GFP, expressed and functionally analysed. The results revealed that the GFP-SH3 protein was able to bind to *S. aureus* cells, which is in accordance with previous studies that reported that SH3 is the binding domain of endolysins in general^29,39,51^ and of *Staphylococcus* phage endolysins in particular^39,52^. Interestingly, GFP-AMI_SH3 protein, which contains the amidase and SH3 domains, presented a higher binding efficiency to *S. aureus* cells than the GFP-SH3, suggesting that the amidase domain contributes to a more efficient binding of the SH3. This might be explained by the PGRP domain that was predicted in the same region of the Amidase_2, and that comprises conserved pattern recognition molecules that bind to bacterial peptidoglycan^53^. However, when the Amidase_2 domain (GFP-AMI) was used alone with *S. aureus* cells did not present any binding capacity. A previous study also showed that the amidase domain improved the binding affinity of a staphylococcal CBD to target bacterial cells, which resulted in an enhanced lytic activity of the endolysin^54^.

The specificity and sensitivity assays performed with the GFP-AMI_SH3 revealed that this protein was able to bind to all *Staphylococcus* spp. tested. This constitutes an advantage of this biorecognition molecule once these pathogens have been frequently isolated from ICU infections^55^, being the CoNS and *S. aureus* responsible for 20.5% and 8.7% of ICU-acquired BSIs in Europe, respectively^55^. Other authors also reported a binding spectrum at the genus level for other CBDs from *Staphylococcus* endolysins^39,52,56^. Some studies suggested that CBDs recognize conserved binding ligands such as the glycine-rich inter-peptide bridge common to most staphylococcal strains^57–59^, as it has been reported for the SH3b-like cell wall targeting domain of lysostaphin^60^, which can explain their broad host range. Curiously, the GFP-AMI_SH3 protein demonstrated some binding affinity to two *Enterococcus* strains and one *S. pneumoniae* strain. Although the binding affinity of GFP-AMI_SH3 was not high and only few *Enterococcus* and *Streptococcus* cells were recognized, it can be hypothesized that the GFP-AMI_SH3 binds to ligands of peptidoglycan in the bacterial cell wall that are conserved among these bacteria genera. In fact, although these bacterial species are members of two distinct phylogenetic orders namely *Bacillales* for *Staphylococcus* and *Lactobacillales* for *Streptococcus* and *Enterococcus*, they present peptidoglycan structures relatively similar apart from their cross-bridges that vary widely in composition and length^59^. Other authors also reported a broader binding affinity of endolysins and CBDs, showing recognition to other bacterial species^61,62^, such as the enterococcal PlyV12 endolysin which kills enterococcal, streptococcal and staphylococcal strains^62^ and the *Staphylococcus* SH3b domain of endolysin LysF1 that binds to *Staphylococcus* and *Streptococcus* species^58^. We can also hypothesise that the amidase domain plays a role in the binding of SH3b to other bacterial species. Further investigations into the nature of the SH3 receptor molecules and the amidase domain are needed, which may be important for the understanding of the binding mechanisms of other endolysin CBDs as well.

The endolysin E-LM12 demonstrated capability to lyse all the *Staphylococcus* strains tested, including CoNS. These results are in agreement with other studies that report that *Staphylococcus* phage endolysins present a broad lytic activity covering all the *Staphylococcus* genus and commonly their spectrum is similar to the one from their CBD^56,63^. Also, the amino acid sequence of E-LM12 is very conserved, being closely related to several staphylococcal endolysins, namely LysK, which was described to be effective against *S. aureus* and CoNS^64^.

Regarding the LM12 phage, it exhibited a lytic effect against most of the *Staphylococcus* spp., although in some strains “lysis from without” was observed, which indicates the absence of phage infection^50^. On the other hand, the GFP-AMI_SH3 protein bound to all the staphylococcal strains tested, demonstrating its higher potential as a recognition probe for *Staphylococcus* spp. when compared with the phage particle.

Regarding the flow cytometry assays, the results indicate that this methodology was able to specifically detect all the *Staphylococcus* species tested and no false positives or false negatives were obtained. In this study, the developed assay revealed to be capable of detecting 10^5^ CFU mL^−1^ of *S. aureus* cells without enrichment, which was expected since the minimal concentration of bacteria detectable by flow cytometry is reported to be in the range of 10^3^ to 10^4^ CFU mL^−1^, using viability stains^65^. The detection around these limits affects the precision of the measurements due to the relatively poor analytical sensitivity^65^.

The flow cytometry assay had to be adapted and optimized for the detection of *Staphylococcus* in blood samples since the number of microorganisms present in circulation during a BSI ranges from 1 to 100 CFU mL^−1^ ^26,27^. Therefore, a sample preparation method was designed to include a pre-enrichment step, allowing the effective detection of 1–5 CFU of *Staphylococcus* in 10 mL of blood. The enrichment procedure also assures the detection of viable cells^66^, preventing the occurrence of false positives. Moreover, the developed method avoided the occurrence of positive signals derived from the autofluorescence of blood components^26,43,67^ and revealed a good analytical reproducibility with results that were easily and objectively interpreted.

The conventional culture methods for the detection of *Staphylococcus* in blood are time-consuming and laborious. In fact, the mean time to blood culture positivity of *S. aureus* is reported to be between 12 to 16 hours^68–70^ and after this, the phenotypic tests must be implemented to identify the specific pathogen which takes about 12 to 36 hours^17^. The assay described herein took less than 2 hours to perform after a pre-enrichment step, and therefore provides a rapid, highly sensitive and reliable methodology for the detection of pathogens in blood, outstanding the standard method of BSI diagnosis. Moreover, in this case, the extensive time required for bacterial enrichment was due to the initial bacterial load of 1 CFU per 10 mL of blood. Considering that the time to blood culture positivity is inversely proportional to the initially inoculated concentration^71^, the turnaround time of our assay can be significantly reduced if a higher concentration of *Staphylococcus* is present in patient blood samples.

Very few phage-based assays have been described in the literature for the detection of bacteria directly from blood^72–74^. Our implemented method presents several advantages over others which require extensive sample pre-treatment^74–76^, genetic manipulation of phage genomes^73^, complex instrumentation^22,75–77^, expensive biorecognition molecules^22,43,77,78^ or entail nucleic acids extraction to complete the detection^74–76^.

In summary, in this study, we have identified the C-terminal fragment of a *Staphylococcus* endolysin that presents a high binding affinity and specificity, which is composed not only of the obvious SH3b domain but also of the Amidase_2 domain. Moreover, we developed a novel and highly reliable endolysin-based flow cytometry assay that allows the rapid and specific detection and identification of *Staphylococcus* spp. from blood cultures.

The designed flow cytometry assay presents several advantages over other bacterial detection methods described in the literature, such as the high specificity rendered by the use of specific phage proteins, simple and inexpensive sample preparation, and the possibility to be tailored to detect other pathogens by using target-specific CBDs. Furthermore, the method can be adapted to a multiplex assay, if specific CBDs are fused with different coloured fluorescent proteins, allowing the simultaneous detection of pathogens prevalent in BSIs. The developed method can also be easily adapted to different clinical samples and types of bacterial infections, by adjusting the sample preparation methodology.

This work is a proof-of-concept that this novel phage endolysin-based flow cytometry assay can be employed in blood samples for the specific detection of bacterial cells. We envisage its validation in blood samples from septic patients in order to be implemented in a clinical environment.

## Material and Methods

### Bacterial strains and growth conditions

The bacterial collection strains were obtained from the Spanish Type Culture Collection (CECT) and clinical isolates were provided by the Hospital of Braga (Portugal). To complete the binding spectrum, additional Gram-positive and Gram-negative species from private collections were used. This accounts for a total of 42 strains used (Table 1), including 12 *S. aureus*, 10 non-*S. aureus* staphylococcal strains, and 9 representative Gram-positive and 11 Gram-negative bacteria.

The strains were routinely grown in Tryptic Soy Broth (TSB) (VWR Chemicals) and in Luria Bertani (LB) (Liofilchem) at 37 °C under agitation (120 rpm) or in solid plates, obtained by adding 12 g L^−1^ of agar (Liofilchem). *Escherichia coli* BL21 (DE3) cells were grown in LB supplemented with kanamycin 50 μg mL^−1^ (NZYTech). The bacterial growth was determined by measuring the optical density at 620 nm (OD_620_ nm) in 96-well plates (Orange Scientific) using a Multiskan™ FC Microplate Photometer (Thermo Fisher Scientific).

### ***In Silico*analysis of staphylococcal endolysin**

The E-LM12 (NCBI Reference Sequence: AUV56903.1) was previously identified as an endolysin belonging to the *S. aureus* vB_SauM-LM12 phage (NCBI Reference Sequence: MG721208.1)^46^. The sequence was analysed through BLAST^79^ to find possible homologous sequences. The search for functional domains was carried using Pfam^80^, HHpred^81^ and InterProScan^82^. The molecular weight and isoelectric point of the proteins were calculated using the Compute pI/Mw program ExPASy^83^.

### Cloning of E-LM12 endolysin domains

Primers containing specific restriction cloning sites (Table 2) were designed to amplify different fragments of the E-LM12 C-terminus (Fig. 1) and insert them into the pET_GFP plasmid^32^ (plasmid pET28a(+) from Novagen with the *Aequorea coerulescens* GFP gene inserted between the NdeI and BamHI restriction sites). Three different fragments (Supplementary Material S3) were amplified by PCR: a fragment containing only the Amidase domain (referred as GFP-AMI); another containing only the SH3 domain (GFP-SH3); and one containing both domains (GFP-AMI_SH3). Primer melting temperatures were calculated using OligoCalc^84^. The fragments were amplified with Phusion DNA Polymerase (Thermo Fisher Scientific) with vB_SauM-LM12 phage as template DNA. The PCR protocol consisted of an initial denaturation step at 98 °C for 30 s; followed by 35 cycles of: denaturation at 98 °C for 10 s, annealing at 60 °C for 30 s and elongation at 72 °C for 1 min; and a final extension step at 72 °C for 10 min. The products were then digested with the restriction enzymes (Table 2), inserted into the pET_GFP (in order to fuse them with the GFP upstream) and ligated with the T4 ligase (New England Biolabs) to obtain the different constructions. The ligation was transformed into competent *E. coli* BL21 (DE3). Colonies were screened through colony PCR and positives were used for plasmid extraction and further confirmation through Sanger sequencing.

**Table 2:** Oligonucleotide primers used for cloning and the respective restriction enzyme used. Enzyme restriction sites are underlined.

Primer Name	Oligonucleotide sequence (5′→3′)	Restriction site
pET_GFP-AMI.fw	CCGCCGCATATGGAATTCAAAAAAGAAACAGCTAAGAAAAGTGCAAGT	EcoRI
pET_GFP-AMI.rv	CCGCCGCTCGAGTCATACAGTAGAACTTGAAGTTCCATTACT	XhoI
pET_GFP-SH3.fw	CCGCCGCATATGGAATTCAGACCATCACAAGCAATAATGAATAAATTAAA	EcoRI
pET_GFP-SH3.rv	CCGCCGCTCGAGTTAACCTTTGAATACACCCCAGG	XhoI

### Expression and purification of GFP fused proteins

*E. coli* BL21 cells harbouring each recombinant plasmid were grown at 37 °C in LB medium supplemented with 50 μg mL^−1^ of kanamycin until reaching an OD_620nm_ = 0.5. Recombinant protein expression was induced with 1 mM isopropyl-β-D-thiogalactopyranoside (IPTG, Sigma-Aldrich), followed by incubation overnight on an orbital shaker at 16 °C, 120 rpm. In the case of *E. coli* BL21 pET28a‐GFP, the expression was carried out at 37 °C, 120 rpm, overnight.

Cells were harvested by centrifugation (9,000 × g, 15 min, 4 °C) and further resuspended in phosphate lysis buffer (20 mM sodium dihydrogen phosphate, 500 mM sodium chloride, pH 7.4). Cells were submitted to freezing (−80 °C) and thawing (30 °C) cycles (3 times) and disrupted by sonication at 40% power (Ultrasonic Processor, Cole Parmer CP 750) for 5 min (30 s ON / 30 s OFF). The cells were centrifuged (9,000 × g, 15 min, 4 °C) to recover the supernatant containing soluble proteins, which was passed through a nickel - nitrilotriacetic acid (Ni-NTA) column (Thermo Fisher Scientific). After washing steps (lysis buffer supplemented with 30 mM imidazole), the proteins were eluted with 300 mM imidazole. The purified proteins were analysed through SDS-PAGE (12% (w/v) acrylamide), followed by Blue Safe staining (NZYTech). The purified proteins were concentrated and dialyzed against 0.1 M phosphate buffer pH 7.2 (PB) using the centrifugal filters Amicon Ultra − 0.5 mL MWCO 10 KDa (Merck Millipore) and stored at 4 °C. Protein concentration was determined using the BCA Protein Assay Kit (Thermo Fisher Scientific) with bovine serum albumin (BSA) as standard.

### Functional analysis of the E-LM12 endolysin C-terminus and specificity assays by epifluorescence microscopy

The binding ability of the different constructions of the E-LM12 endolysin C-terminus fused to GFP (GFP-AMI, GFP-AMI_SH3 or GFP-SH3) was inferred by fluorescence microscopy observations of *S. aureus* Sa12 cells after incubation with the fused proteins. Briefly, bacterial cells were grown in 10 mL of liquid LB or TSB at 37 °C until mid-log phase (OD_620nm_ = 0.6) and then the culture was centrifuged for 5 min at 6,000 × g, followed by resuspension in 10 mL of PB. A volume of 500 µL of each bacterial suspension was centrifuged at 6,000 × g for 5 min. The pellet was resuspended in 20 µL of purified GFP fused protein (at a concentration of 5 µM) and incubated for 30 min at room temperature. The cells were washed three times with PB by centrifugation (6,000 × g, 5 min) to remove the unbound protein. The washed pellet was resuspended in 10 µL of PB and observed at the epifluorescence microscope equipped with U-RFL-T light source (Olympus BX51, Magnification 1,000×) in bright field and under the FITC filter (Excitation BP 470–490 nm; Emission: LP 516 nm). Control samples of bacterial cells without addition of the recombinant proteins were prepared simultaneously. GFP alone was used as a negative control.

For the assessment of the specificity and sensitivity of GFP-AMI_SH3, bacterial species listed in Table 1 were incubated with this protein, following the same procedure described above and observed at the epifluorescence microscope.

### Phage lytic spectra

The host range specificity of the LM12 phage was screened against all strains listed in Table 1. Bacterial lawns were made on Tryptic Soy Agar (TSA) plates by mixing 100 µL of exponential-phase cell cultures of each strain with 3 mL of TSA soft overlays (TSB with 0.4% (w/v) of agar). The bacterial lawns were spotted with 10 µL drops of the phage solution (10^9^ PFU mL^−1^). LM12 phage lysis was assessed by adding 10 µL drops of serial 10-fold dilutions of phage stock to the bacterial lawns. After 16–18 hours incubation at 37 °C, plates were inspected for lysis zones and results were scored as: “positive” for clear lysis areas; “negative” for the absence of lysis areas; and “lysis from without” (LFW) when clear areas were observed with high phage titers but no plaques appeared when lower phage concentrations were spotted^50^.

### E-LM12 lytic activity

The E-LM12 lytic activity was carried out by the spot lysis test against all *Staphylococcus* strains listed in Table 1. Bacterial lawns were made on TSA plates by mixing 100 µL of exponential-phase cell cultures (OD_620 nm_ = 0.4) of each strain with 3 mL of TSA soft overlays. The bacterial lawns were spotted with 10 µL drops of E-LM12 at different concentrations (2, 5, 8, 10 and 15 µM).

### Flow cytometry assays

For the flow cytometry assays, the three different C-terminus domains of E-LM12 (GFP-AMI, GFP-AMI_SH3 or GFP-SH3) were initially tested against *S. aureus* Sa12 cells. Bacterial cells were grown in 10 mL of liquid LB or TSB at 37 °C until mid-log phase (OD_620nm_ = 0.6). Briefly, 1 mL of each bacterial suspension was centrifuged at 6,000 × g for 5 min and the pellet resuspended in 20 µL of purified GFP fused protein at a concentration of 5 µM and incubated for 30 min at room temperature. The cells were washed twice with PB by centrifugation (6,000 × g for 5 min) to remove the unbound protein and cells were resuspended in 200 µL. The samples were analysed using an EC 800 flow cytometer equipped with a diode blue laser (excitation at 488 nm) (Sony Biotechnology). A total of 45,000 events were acquired with a sample flow rate of 10 μL min^−1^. The fluorescence was detected through a 525/50 nm band-pass filter on the channel FL1. Data analysis was performed using FCS Express 6 RUO software (De Novo Software).

For the evaluation of the specificity and sensitivity of GFP-AMI_SH3, this protein was incubated with the bacterial strains listed in Table 1, following the same procedure described above. Control samples of bacterial cells without addition of the recombinant protein were prepared simultaneously. The detection limit of the method was assessed by using different concentrations of *S. aureus* Sa12 ranging from 1 × 10^4^ to 1 × 10^8^ CFU mL^−1^, following the procedure described above. The bacterial loads were quantified by flow cytometry and by Colony-forming units (CFU) counting.

#### **Detection of*S. aureus*in spiked blood samples**

For the detection of *S. aureus* in artificially seeded blood, 10 mL of defibrinated horse blood (Thermo Fisher Scientific) was mixed with 90 mL of TSB culture medium. The blood sample was then inoculated with a concentration of 1 to 5 CFU mL^−1^ of *S. aureus* Sa12 and incubated 16 hours at 37 °C, 120 rpm. A non-inoculated sample was prepared in parallel and exposed to the same conditions to be used as a control. One millilitre of each sample was recovered, diluted 10 times in sterile water to promote the lysis of erythrocytes by osmotic stress, centrifuged at 6,000 × g for 10 min, washed twice with PB and then incubated with GFP-AMI_SH3 (at a concentration of 10 µM), following the procedure described before. To confirm the specificity of the assay, the strain *Klebsiella pneumoniae* 35 was used as a negative control, following the same procedure described for *S. aureus*. Moreover, since by epifluorescence microscopy, GFP-AMI_SH3 showed a weak binding to *E. faecalis* LMV-0-39 and *E faecium* LMV-0-42, these strains were tested by flow cytometry and *E. faecalis* CECT 184 was used as a negative control.

### Statistical analysis

All results were analysed by One-way ANOVA test. The data are presented as means and standard deviations. Differences between samples were considered statistically significant for p-values lower than 0.05.

### Accession number

The NCBI Reference Sequence of the endolysin E-LM12 is AUV56903.1.

### Ethical approval

This article does not contain any studies with human participants or animals performed by any of the authors.

## Supplementary information

Supplementary Dataset 1.
