# Supplementary material for: A novel flow cytometry assay based on bacteriophage-derived proteins for Staphylococcus detection in blood
Source: Sci Rep. 2020 Apr 10;10:6260. doi: 10.1038/s41598-020-62533-7 (PMC7148305; doi:10.1038/s41598-020-62533-7)
Supplement: Supplementary file 1 — Supplementary Dataset 1. [file 41598_2020_62533_MOESM1_ESM.pdf]

A novel flow cytometry assay based on bacteriophage-derived proteins for *Staphylococcus* detection in blood

Susana P. Costa[a, b], Nicolina M. Dias[a], Luís D. R. Melo[a], Joana Azeredo[a], Sílvio B. Santos[a], Carla M. Carvalho[*a, b]

[a] Centre of Biological Engineering, University of Minho, Campus de Gualtar, 4710-057, Braga, Portugal
[b] Department of Nanoelectronics Engineering, Nanodevices Research Group, International Iberian Nanotechnology Laboratory, Av. Mestre José Veiga s/n, 4715-330, Braga, Portugal

*Corresponding author: carla.carvalho@inl.int

Supplementary Material S1 - Assessment of the binding affinity of GFP-AMI_SH3 protein after decoration of *S. pneumoniae* R6st and *E. faecium* LMV-0-42 cells

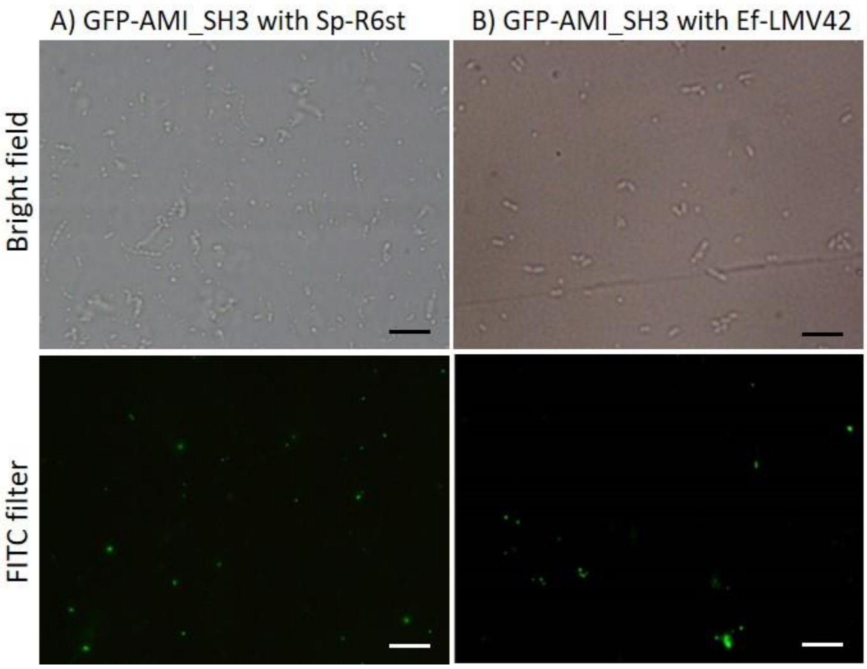

Figure S1 - Fluorescence microscopy images of *S. pneumoniae* R6st (A) and *E. faecium* LMV-0-42 (B) after incubation with GFP-AMI_SH3 protein. Observations were made in bright field and under FITC filter with the same exposure time to detect the presence/absence of fluorescing cells. Scale bar represents 10 µm

Supplementary material S2 - Graphical representation of blood assays performed for *Enterococcus* species.

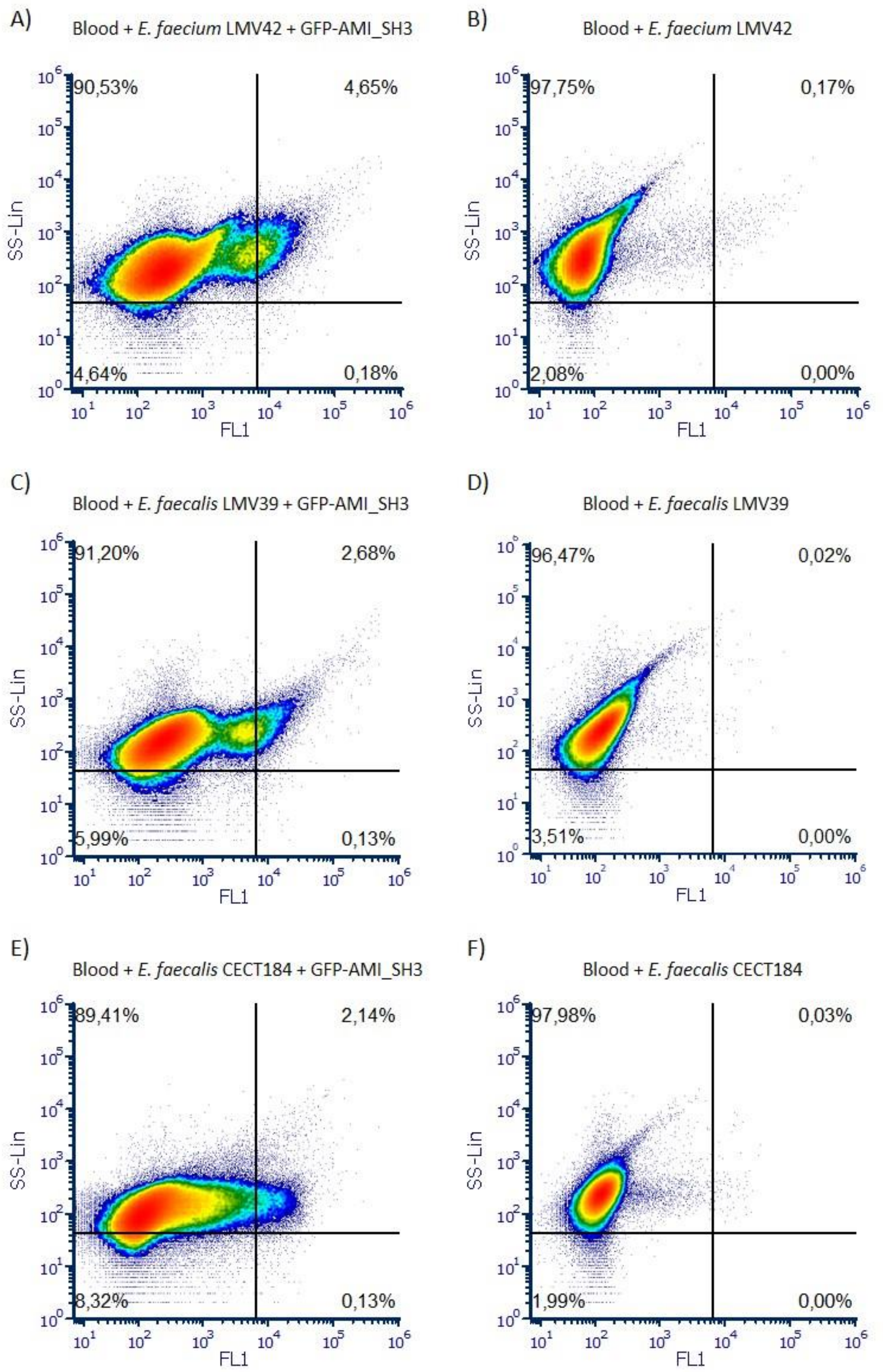

Figure S2 - Graphical representation of blood samples analysis by flow cytometry. Representative dot plots showing side scattering (SS) and green fluorescence intensity (FL1) of blood culture with *E. faecium* LMV-0-42 (A), *E. faecalis* LMV-0-39 (C) and *E. faecalis* CECT184 (E) incubated with GFP-AMI_SH3 protein; and the respective blood cultures with unlabelled *E. faecium* LMV-0-42 (B), *E. faecalis* LMV-0-39 (D) and *E. faecalis* CECT184 (F).

Supplementary Material S3 – Gene sequences

Ami (Amidase domain, 633 bp)

AAAAAAGAAACAGCTAAGAAAAGTGCAAGTAAAACTCCTGCACCTAAAAA
GAAAGCAACACTAAAAGTTTCTAAGAACCATATTAACTATACAATGGATAA
ACGTGGTAAGAAACCTGAAGGAATGGTAATACACAACGATGCGGGTCGTTC
TTCAGGACAACAATATGAAAACTCTTTAGCTAAAGCCGGTTATGCTAGATA
CGCTAATGGTATTGCTCACTACTATGGTTCAGAAGGTTATGTATGGGAAGCA
ATAGATGCTAAGAATCAAATTGCTTGGCATACAGGTGATGGAACAGGAGCT
AACTCAGGTAACTTTAGATTTGCAGGTATTGAAGTCTGTCAATCAATGAGTG
CTAGTGATGCTCAATTCCTTAAAAACGAACAAGCAGTATTCCAATTCACAGC
AGAGAAATTCAAAGAATGGGGTCTTACTCCTAACCGTAAGACTGTAAGATT
GCACATGGAGTTCGTTCCTACAGCTTGTCCTCACCGTTCAATGGTACTTCAT
ACAGGATTCAATCCAGTAACTCAAGGAAGACCATCACAAGCAATAATGAAT
AAATTAAAAGATTATTTCATTAAACAAATTAAAAACTACATGAGTAATGGA
ACTTCAAGTTCTACTGTA

SH3 (SH3 domain, 444 bp)

AGACCATCACAAGCAATAATGAATAAATTAAAAGATTATTTCATTAAACAA
ATTAAAAACTACATGAGTAATGGAACTTCAAGTTCTACTGTAGTTAAAAAA
GGTAAAACAAGTAGTGCAAGTACACCGGCAACTAGACCTGTAACAGGTTCT
TGGAAAAAGAACCAGTTCGGAACTTGGTACAAACCGGAATCGGCAACGTTT
GTTAATGGTAACCAACCTATAGTAACTAGAATAGGTTCTCCATTCTTAAATG
CTCCAGTAGGAGGTAACCTCCCTGCAGGTGCTACAATTGTATATGACGAGG
TAGCAATCCAAGCAGGTCATATTTGGATTGGTTATAACGCTTATAATGGTAA
CAGAGTATATTGCCCTGTTAGAACATGTCAAGGTGTTCCACCAAACCATATA
CCTGGTGTTGCCTGGGGTGTATTCAAAGGTTAA

AMISH3 (Amidase and SH3 domains, 984 bp)

AAAAAAGAAACAGCTAAGAAAAGTGCAAGTAAAACTCCTGCACCTAAAAA
GAAAGCAACACTAAAAGTTTCTAAGAACCATATTAACTATACAATGGATAA
ACGTGGTAAGAAACCTGAAGGAATGGTAATACACAACGATGCGGGTCGTTC
TTCAGGACAACAATATGAAAACTCTTTAGCTAAAGCCGGTTATGCTAGATA
CGCTAATGGTATTGCTCACTACTATGGTTCAGAAGGTTATGTATGGGAAGCA
ATAGATGCTAAGAATCAAATTGCTTGGCATACAGGTGATGGAACAGGAGCT
AACTCAGGTAACTTTAGATTTGCAGGTATTGAAGTCTGTCAATCAATGAGTG
CTAGTGATGCTCAATTCCTTAAAAACGAACAAGCAGTATTCCAATTCACAGC
AGAGAAATTCAAAGAATGGGGTCTTACTCCTAACCGTAAGACTGTAAGATT
GCACATGGAGTTCGTTCCTACAGCTTGTCCTCACCGTTCAATGGTACTTCAT
ACAGGATTCAATCCAGTAACTCAAGGAAGACCATCACAAGCAATAATGAAT
AAATTAAAAGATTATTTCATTAAACAAATTAAAAACTACATGAGTAATGGA
ACTTCAAGTTCTACTGTAGTTAAAAAAGGTAAAACAAGTAGTGCAAGTACA
CCGGCAACTAGACCTGTAACAGGTTCTTGGAAAAAGAACCAGTTCGGAACT

TGGTACAAACCGGAATCGGCAACGTTTGTTAATGGTAACCAACCTATAGTA
ACTAGAATAGGTTCTCCATTCTTAAATGCTCCAGTAGGAGGTAACCTCCCTG
CAGGTGCTACAATTGTATATGACGAGGTAGCAATCCAAGCAGGTCATATTT
GGATTGGTTATAACGCTTATAATGGTAACAGAGTATATTGCCCTGTTAGAAC
ATGTCAAGGTGTTCCACCAAACCATATACCTGGTGTTGCCTGGGGTGTATTC
AAAGGTTAA
